# A case study for the use of medical cannabis in generalized anxiety disorder

**DOI:** 10.15190/d.2019.5

**Published:** 2019-06-12

**Authors:** Chad Walkaden

**Affiliations:** Counselling and Consulting Services, New South Wales, 2099, Australia

**Keywords:** Mental health, anxiety, medical cannabis, healthcare, quality of life, integrative medicine.

## Abstract

Despite the increasing prevalence and acceptance of the medical cannabis use among the general public, the evidence required by physicians to use cannabis as a treatment is generally lacking. Research on the health effects of cannabis and cannabinoids has been limited worldwide, leaving patients, health care professionals, and policymakers without the evidence they need to make sound decisions regarding the use of cannabis and cannabinoids. This case study outlines an intervention that involved a patient integrating medical cannabis into her treatment to better manage a generalized anxiety disorder and the debilitating symptoms of vertigo. This case demonstrates how the patient drastically improved her quality of life and reinforces the need for more rigorous testing on the use of medical cannabis to support patients and better manage the symptoms associated with their medical conditions.

## INTRODUCTION

Despite the increasing prevalence and acceptance among the general public, the prescription of cannabis for treating a range of medical conditions continues to be viewed with caution^1^.

In the USA, there are 34 states that have legalized medical cannabis^2^. These legislative changes equate to over 59 million people that are legally using cannabis across the country^3^. While this represents roughly 18% of the population, the number could be considerably larger if the prescription of cannabis as a treatment was supported by physicians and the associations they are part of. Among other points, the claims against the prescription of medical cannabis include recommendations that approved conventional drugs are undertaken before cannabis products are used for treatment^4^ and that medical cannabis undergoes the same rigorous approval process of other medications prescribed by physicians, including randomized, placebo-and active-controlled trials^5^. 

Notwithstanding the requirement for evidencebased information on the health effects of cannabis and cannabinoids, a conundrum exists whereby the federal government has not legalized cannabis and continues to enforce restrictive policies and regulations^6^. As a result, research on the health effects of cannabis and cannabinoids has been limited in the United States, leaving patients, health care professionals, and policymakers without the evidence they need to make sound decisions regarding the use of cannabis and cannabinoids^6^. This lack of evidence-based information is the cause of the growing need to understand the role medical cannabis can have in improving the health outcomes for patients with complex medical conditions.

## CASE HISTORY

 a. The 88-year-old female presented to seek support around a decline in her quality of life and challenges with her emotional and psychological well-being. 

b. Her challenges were primarily associated with managing a generalized anxiety disorder and the debilitating symptoms of vertigo. She described experiencing “wonky days” that consisted of intense dizziness, extreme nausea, imbalance, and worry about what is going to happen if her symptoms worsened. She described a consistently low mood, discomfort in her daily life and a belief that she was losing control of her life. 

c. This problem commenced 24 months earlier and was compounded by an anxiety about the continual impact of these concerns if not resolved. The patient was also experiencing ongoing grief associated with losing her late husband of 68 years five years earlier. 

d. The debilitating symptoms were consistent over 24 months before the intervention. In a typical week, the symptoms consisted of “wonky days” that had a frequency of three to four times per week at an intensity where her quality of life was impacted to a score of eight out of 10. 

e. Initially, the benzodiazepine Kalma, was prescribed to treat her anxiety. However, the patient stopped taking this after two days due to incessant shaking. The patient was then prescribed another benzodiazepine, Xanax. She reported that she did not consume this drug due to concern that she would have a reaction similar to what she experienced with Kalma, since the active ingredient is the same, alprazolam. 

f. Relevant history includes lymphatic cancer and ongoing grief from the loss of her late husband. There was no relevant family history. 

g. The assessment revealed a white Australian female that was the mother of three adult daughters, several grandchildren and one great-grandson. She presented as friendly and was easy to engage and establish rapport with. She described strong family, peer and community relationships that provided practical and emotional support. Before these challenges, she provided an overview of a fulfilling, healthy, stable, and happy adult life. She was able to articulate her challenges with her independence, mobility and the uncertainty about the continual impact of debilitating symptoms for herself and her family. She expressed desperation to improve her deteriorating health that was a significant risk to her quality of life and overall survival. From the onset of these challenges, she had sought support from her medical team and this care continued throughout the intervention.

## METHODS AND RESULTS

a. The patient undertook a mental health intervention in a format that consisted of weekly individual consulting sessions for the initial six weeks before it extended to individual sessions once every two weeks for a further 10 weeks. 

b. The mental health intervention was based on a humanistic methodology that combines a reparative approach most commonly associated with counselling, with the addition of exercise, meditation and other lifestyle components incorporated into her treatment plan. 

c. Throughout the intervention, the patient maintained a written record of her reflections and progress. In the beginning, she described her circumstances by saying “I just want the wonkiness to stop”, “I have lost my capacities” and “I have lost my independence”. 

d. During the initial four weeks, the patient was supported to reframe her beliefs about her changing capacities and she was encouraged to explore the ongoing grief that was associated with the loss of her late husband. 

e. By the fifth week, a protocol was designed for the patient to complete on a daily basis. This protocol involved the patient being instructed to replace known problematic times where her symptoms spiked with guided strategies that targeted the compounding impact of the beliefs associated with her debilitating symptoms. While the patient reported that the protocol resulted in reductions in the frequency of her symptoms to a maximum of one to two days per week, the intensity of her symptoms when occurring were still scored at an eight out of 10. 

f. In the sixth week, the patient reported that she had sourced her own medical cannabis oil that was made from the OG Kush strain of cannabis. She reported that she maintained a daily dose of 2 ml of a medical cannabis that contained Tetrahydrocannabinol or more commonly known as THC. The patient reported that, after incorporating the cannabis oil into the protocol, the intensity of her symptoms ceased and that she could be free from any symptoms for a period of at least two weeks. 

g. By the tenth week, the patient reported significant improvements to her quality of life. She reported that the “wonky days” had almost been completely eliminated and she was continuing to adhere to her protocol that involved recommencing daily physical exercises and meditation practices. At this stage of the intervention, the patient reported that “life is as good as it gets”. 

h. In the fifteenth week, the patient stopped using medical cannabis due to the inability to access more. She reported that the “wonkiness” returned and that she was again suffering from daily nausea, dizziness and an imbalance to her mobility. 

i. In the sixteenth and final week of the intervention, the patient had reintegrated medical cannabis into her daily protocol and she had been able to once again regain her quality of life through the cessation of her symptoms. 

j. In a review three months after the intervention, the patient reported that she had now resumed the daily protocol that includes taking a dose of 2 ml of medical cannabis in the morning. The patient reported that, since finishing the intervention, she experienced a four-week period where she was unable to access a further supply of medical cannabis. She reported that, during the four weeks, her physical health, mental health and quality of life deteriorated. These deteriorations included a life alert system being installed in her home by her family due to concern about her safety. The patient also reported that she had informed her medical team about her daily consumption of a medical cannabis oil. She reported that her medical team could not prescribe her a cannabis oil but that they supported and encouraged her to continue the cannabis oil as a daily treatment. The patient did not have a scheduled annual blood test due to her fear of needles. Thus, no blood tests have been completed to identify any chemical changes that were associated with her introducing medical cannabis into her life.

## DISCUSSION

While it is clear that the popularity of cannabis is increasingly for the general public, there is a clear need to gain more scientific knowledge about the associated benefits and risks of cannabis for patients with medical conditions.

Undoubtedly, there are many unsubstantiated claims about the use of cannabis for treating several medical conditions. However, there is scientific evidence that compounds naturally found in marijuana have therapeutic benefit for symptoms of diseases such as HIV/AIDS, multiple sclerosis, cancer and specific forms of epilepsy^6^. More specifically for anxiety, scientific evidence is also showing cannabis is expected to relieve tension and help young females relax^7^. 

Although the future role that medical cannabis will play in healthcare is unknown, this case demonstrates how the patient was able to significantly benefit from the introduction of medical cannabis into her mental health intervention for the treatment of vertigo and a generalized anxiety disorder. In this case, the benefits for the 88-year-old patient using medical cannabis as a treatment in the both the short term and longer-term far outweighed the potential risks that may require consideration for children or adolescents^8^. 

This case demonstrates how the patient was able to use medical cannabis to reduce the debilitating symptoms associated with her vertigo to drastically improve her quality of life. A pattern seems evident for the patient where her symptoms for vertigo fluctuated according to whether or not she was able to access medical cannabis. This case also highlights the legislative conundrum for patients, physicians and government^9^. Despite her medical team supporting her continual use of medical cannabis, she was not able to receive a prescription. As a result, she accessed medical cannabis from an illegal and unregulated source. While the patient reported to trust this source and only use medical cannabis according to her daily protocol, there are potential safety risks associated with her self-medicating cannabis to combat her symptoms and not having accurate information about dosing, consistency and quality of the cannabis.

## CONCLUSION

This case is a preliminary finding and reinforces the need for more rigorous testing on the application of medical cannabis to be used as a treatment for different medical conditions, including generalized anxiety disorder.

## KEY POINTS 

**◊ This case study outlines the potential benefit of medical cannabis for the management of generalized anxiety disorder and debilitating symptoms of vertigo ◊ The patient drastically improved her quality of life ◊ There is a need for more rigorous testing on the use of medical cannabis to support patients and better manage their symptoms ◊ Also, there are significant safety risks posed for the patients if they access medical cannabis from an unregulated source and use it without supervision.**
